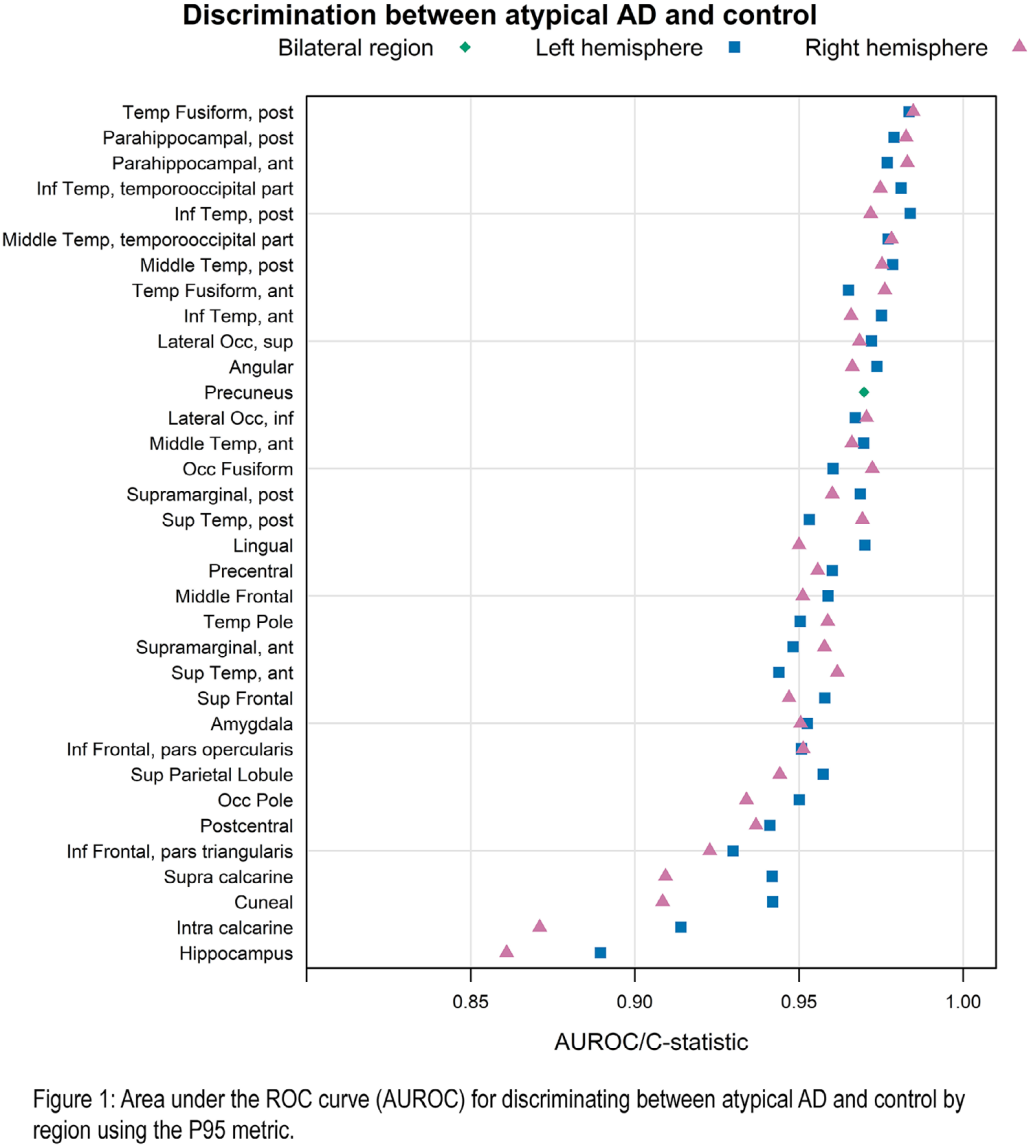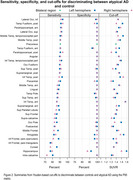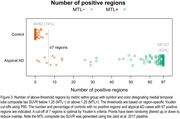# Defining regional tau‐PET positivity cut points in atypical AD

**DOI:** 10.1002/alz70856_106619

**Published:** 2026-01-11

**Authors:** Neha Singh‐Reilly, Stephen D. Weigand, Amanda Tapia, Ryota Satoh, Jonathan Graff‐Radford, Mary M. Machulda, Christopher G Schwarz, Clifford R. Jack, Val J Lowe, Keith A. Josephs, Jennifer L. Whitwell

**Affiliations:** ^1^ Mayo Clinic, Rochester, MN, USA

## Abstract

**Background:**

Atypical clinical presentations of Alzheimer's disease (AD) are associated with heterogeneous patterns of cortical tau‐PET uptake. The medial‐temporal (MTL) meta‐ROI is typically used to determine tau‐PET abnormality in AD, but this approach may not detect focal uptake in atypical patients. Our goal was to develop regional tau‐PET cut‐points and determine whether the number of positive regions better captures positive tau‐PET signal in atypical AD.

**Method:**

127 amyloid‐positive atypical AD (visual=68, language=39, motor=15, others=5) and 62 amyloid‐negative cognitively unimpaired (CU) individuals were recruited by the Neurodegenerative Research Group, Mayo Clinic, and underwent, amyloid‐PET and ^18^F‐AV‐1451(tau)‐PET. Images were parcellated using the modified Harvard‐Oxford atlas and we focused on 67 AD‐specific regions. Rather than using the average SUVR across voxels within a region to quantify uptake, we used the 95^th^ percentile of SUVR values across voxels within a region to capture focal cases. We then used receiver operating characteristic (ROC) methods and a Youden threshold to define regional abnormality. The number of abnormal regions was calculated for each participant and used to discriminate between CU and atypical AD at the individual level.

**Result:**

Across 64 regions, our approach showed excellent (AUROC>0.90) differentiation between atypical AD and CU. The temporal‐fusiform, parahippocampal gyrus and inferior temporal gyrus showed the best differentiation (Figure 1). All 67 regions showed excellent specificity (>85%), with 63 regions showing excellent sensitivity (≥80%). The lateral occipital cortex, temporal‐fusiform and parahippocampal gyrus showed the best specificity and sensitivity (Figure 2). The distribution of the number of tau‐elevated regions is shown in Figure 3. 74% of CU had no positive regions, while 43% of the atypical AD patients had all 67 regions affected. An optimal threshold of 7 tau‐elevated regions classified all CU below, and 123 (97%) atypical AD above threshold. Using a standard approach based on the average SUVR in the MTL meta‐ROI, only 116 (91%) atypical AD patients were tau‐PET positive.

**Conclusion:**

Tau uptake in temporal regions showed the greatest group‐level differentiation. However, examining regional tau‐positivity may offer better sensitivity in atypical AD patients compared to the temporal meta‐ROI, likely due to the presence of focal non‐temporal patterns of uptake.